# Metabolic characteristics of non-obese and obese metabolic dysfunction-associated fatty liver disease in type 2 diabetes mellitus and its association with diabetic peripheral neuropathy and diabetic retinopathy

**DOI:** 10.3389/fmed.2023.1216412

**Published:** 2023-09-27

**Authors:** Si-Wen Dang, Lei Gao, Yu-Jun Li, Ruo Zhang, Jing Xu

**Affiliations:** ^1^Department of Endocrinology, The Second Affiliated Hospital, Xi’an Jiaotong University, Xi’an, China; ^2^International Center for Obesity and Metabolic Disease Research, Xi’an Jiaotong University, Xi’an, China

**Keywords:** non-obese metabolic dysfunction-associated fatty liver disease, type 2 diabetes mellitus, metabolic characteristics, diabetic peripheral neuropathy, diabetic retinopathy

## Abstract

**Aim:**

To assess the metabolic characteristics of non-obese metabolic dysfunction-associated fatty liver disease (MAFLD) compared with obese MAFLD and the relationship of MAFLD with diabetic peripheral neuropathy and diabetic retinopathy in patients with Type 2 diabetes mellitus (T2DM).

**Methods:**

Data were obtained from 536 T2DM patients (355 women, 181 men; age 58.2 ± 12.0 years). We explored the difference in clinical characteristics between obese MAFLD (body mass index ≥25 kg/m^2^) and non-obese MAFLD (body mass index <25 kg/m^2^) in T2DM patients. One-way analysis of variance (ANOVA) was used to compare the means of continuous variables, and the Chi-squared test was used to compare the differences in frequencies of categorical variables. Logistic regression models were adopted to calculate odds ratios.

**Results:**

The prevalence of MAFLD in hospitalized Chinese T2DM patients was calculated to be 42.7%. Both obese and non-obese MAFLD patients had higher levels of body mass index (BMI), waist circumfere nce, triglyceride, alanine aminotransferase, aspar tate aminotransferase, γ-glutamyltransferase, you nger age, higher prevalence of hyperlipidemia and shorter duration of T2DM and lower incidence of diabetic retinopathy, compared with participants with out MAFLD in the same weight group. Uric acid levels were positively correlated with the risk of MAFLD only in non-obese subjects but not in obese subjects. In non-obese patients with T2DM, a negative correlation was found between the prevalence of MAFLD and diabetic retinopathy.

**Conclusion:**

Even in non-obese patients with T2DM, BMI was found to be an independent risk factor for MAFLD. These findings support a more structured, risk-factor-based approach to MAFLD management, particularly in patients with T2DM. Non-obese MAFLD has unique results in metabolic characteristics and the correlation with diabetic retinopathy and diabetic peripheral neuropathy, which should be further explored.

## Introduction

Type 2 diabetes mellitus (T2DM) is reported to affect one in 11 adults and up to 463 million people worldwide, according to the International Diabetes Federation (IDF) (1). Diabetes is an independent risk factor for the development of non-alcoholic fatty liver disease (NAFLD), which can progress to fibrosis, cirrhosis, liver failure, and hepatocellular carcinoma (2, 3). Patients with T2DM and/or obesity have a significantly increased NAFLD prevalence rate of 60–90% (4). While NAFLD doubles the diabetes-associated mortality rates, the presence of T2DM increases the likelihood of NAFLD progression (4, 5). With the growing global prevalence of T2DM, which is projected to exceed 700 million patients by 2045 (1), accurate estimation of clinical risk factors for NAFLD is important to predict those patients who require monitoring for more advanced liver disease or those who may benefit from future disease-modifying agents.

Many studies have investigated the relationship between NAFLD and metabolic risk factors. Evidence suggests that obesity, as measured by body mass index (BMI) or waist circumference, has an impact on the risk of NAFLD (6–8). Lipid abnormalities (low high-density lipoprotein and high triglycerides) and hypertension have both been independently associated with incident NAFLD (8). Elevated liver enzymes such as alanine aminotransferase (ALT), aspartate aminotransferase (AST), and gamma-glutamyl transpeptidase (GGT), in addition to the ALT/AST ratio, are commonly used as surrogate markers of NAFLD (9–11). However, the incidence of NAFLD was found to correlate negatively with microvascular complications of diabetes, i.e., diabetic peripheral neuropathy, diabetic retinopathy, and diabetic nephropathy (12), which was inconsistent with the observations of other studies (13, 14).

Although NAFLD is usually associated with obesity, the subphenotype of lean subjects also presents with NAFLD and is becoming increasingly prevalent. The prevalence of lean NAFLD subjects has been reported to be 10.2% and appears to be more common in Asia (15, 16). It has been proven to be an unrecognized clinicopathologic entity and a frequent cause of cryptogenic liver disease (17). More efforts should be made to halt or reverse the progression of NAFLD in non-obese individuals (18).

Recently, a consensus panel has proposed that NAFLD be renamed metabolic dysfunction-associated fatty liver disease (MAFLD) based on the presence of one of the following three criteria: overweight/obesity, T2DM, and evidence of so-called “metabolic dysregulation” (19). MAFLD can be defined whether with or without alcohol consumption or other other concomitant liver diseases, which differs from the previous term NAFLD. Because MAFLD is not a widely used term in the scientific literature, most published data focus on NAFLD. The most recent prevalence and risk factors of the newly defined MAFLD are rare.

The present study determined the prevalence and some risk factors for MAFLD in patients with T2DM and evaluated their correlations with diabetic complications. Differences in the prevalence of MAFLD in patients with T2DM between overweight/obese and lean (categorized by BMI), in addition to the clinical and biochemical characteristics of the condition, are discussed.

## Materials and methods

### Study population

Participants in this cross-sectional study were recruited from patients who visited the Second Affiliated Hospital of Xi’an Jiaotong University in Xi’an, Shaanxi Province, from August 2016 to July 2018. A total of 536 T2DM patients aged 18–89 years with comprehensive anthropometric measurements, clinical examinations, abdominal ultrasound, and questionnaires were included in the study. This study protocol was approved by the Epidemiology Ethics Committee of the Second Affiliated Hospital of Xi’an Jiaotong University, Xi’an.

T2DM was defined based on a self-reported history of diabetes as previously determined by a healthcare professional or fasting plasma glucose ≥ 126 mg/dL (7.0 mmol/L). Exclusion criteria included: (I) type 1 diabetes, gestational diabetes, or special types of diabetes; (II) acute complications of diabetes, severe infections, end-stage renal disease, blood disease and/or other complications due to metabolic disorder; (III) recent change (≥10%) in body weight; (IV) use of medications that may cause fatty liver, such as glucocorticoids, synthetic estrogens, olanzapine; (V) ALT, AST, or GGT levels greater than three times normal; (VI) diseases/conditions that affect glycolipid metabolism, including total parenteral nutrition, inflammatory bowel disease, anterior hypopituitarism, hyperthyroidism, Cushing’s syndrome, hemochromatosis; (VII) participation in another clinical trial within the last 30 days.

### Measurement of variables

Weight, height, waist and hip circumference, and systolic and diastolic blood pressures were measured by standard clinical procedures on the very first day of the visit. The body mass index (BMI, kg/m^2^) was calculated as weight (kg) divided by height (m) squared. The waist-to-hip ratio was calculated as the waist circumference divided by the hip circumference. Obesity was defined as having a BMI ≥ 25 kg/m^2^ (20). Fasting blood samples were obtained after an overnight fast for the measurement of alanine aminotransferase (ALT), aspartate aminotransferase (AST), gamma-glutamyl transferase (GGT), total cholesterol, triglycerides, high-density lipoprotein cholesterol, low-density lipoprotein cholesterol, lipoprotein (a), uric acid, and hemoglobin A1c (HbA_1c_). The following variables were extracted from the medical records: age, sex, history of smoking, history of alcohol consumption, and duration of T2DM.

### Diagnostic criteria

The diagnosis of MAFLD in these T2DM patients was based on an ultrasonographic diagnosis of hepatic steatosis. Metabolic syndrome (MetS) in this study, in which all participants were T2DM patients, was defined by the presence of at least two of the following metabolic risk abnormalities: (1) waist circumference ≥ 90/80 cm in men and women; (2) blood pressure ≥ 130/85 mmHg or specific drug treatment; (3) plasma triglycerides ≥ 1.70 mmol/L or specific drug treatment; (4) plasma HDL cholesterol < 1.0 mmol/L for men and < 1.3 mmol/L for women or specific drug treatment. A patient was defined as having hypertension if their blood pressure was higher than 140/90 mmHg or with a history of the disease. If serum triglycerides were ≥ 1.70 mmol/L or serum cholesterol ≥5.18 mmol/L or there was a history of the disease, hyperlipidemia was diagnosed. Diabetic retinopathy was evaluated by experienced ophthalmologists in the presence of retinal hemorrhages, exudates, and macular edema. If required, fluorescein angiography was carried out. Diabetic peripheral neuropathy was diagnosed by a Nerve Conduction Velocity test or the Current Perception Threshold test, or established by the presence of typical symptoms and compatible findings on a neurological examination or a history of treatment for neuropathy.

### Statistical analysis

The distribution of continuous variables was analyzed for normality using the Kolmogorov–Smirnov test. Data are presented as mean ± SD for normally distributed data and as median (25–75%) for non-normally distributed data. To compare characteristics between groups, the unpaired t-test and the Mann–Whitney U test were used for continuous variables, and Fisher’s exact test and the Chi-squared test were used for categorical variables, as appropriate. Logarithmic transformation was performed for non-normally distributed data when necessary. The association of the BMI (an independent variable) with variables (a dependent variable) was assessed by linear regression. Correlation analyses were performed using Pearson’s correlation coefficient. Binary (univariate and multivariate) logistic regression was used to test the potential relationship between variables and the odds ratios of MAFLD in T2DM patients.

## Results

### Characteristics of participants

The baseline characteristics of the study participants according to their MAFLD status are presented in Table 1. All subjects included in our current study were patients with T2DM, of whom 42.7% were also diagnosed with MAFLD, with no statistical difference between men and women (42.8% vs. 42.5%, *p* > 0.05). Participants with MAFLD had higher BMI, waist circumference, hip circumference, waist-to-hip ratio, triglycerides, total cholesterol, ALT, AST, GGT, uric acid levels, and lower high-density lipoprotein cholesterol concentrations than those without MAFLD (*p* < 0.05). Moreover, patients with MAFLD were younger and had a higher proportion of alcohol consumption, hyperlipidemia, central obesity, MetS, and a shorter duration of T2DM than those without NAFLD (*p* < 0.05). It is worth noting that the incidence of diabetic retinopathy and diabetic peripheral neuropathy was lower in participants with MAFLD compared with those without MAFLD (*p* < 0.05). No statistical differences were observed between the groups in blood pressure, low-density lipoprotein cholesterol, lipoprotein (a), HbA_1c_ levels, or smoking history.

**Table 1:** Baseline characteristics of the study participants according to MAFLD status.

Characteristics	All	Without MAFLD	With MAFLD	*p-*value
N	536	307	229	
Sex (female subjects/male subjects)	181/355	104/203	77/152	0.951
Age (years)	59.8 ± 11.8	60.3 ± 12.0	55.4 ± 11.5	<0.001
SBP (mmHg)	133.8 ± 26.6	134.6 ± 25.7	134.2 ± 18.5	0.849
DBP (mmHg)	78.2 ± 13.5	78.0 ± 14.8	80.1 ± 12.0	0.085
BMI (Kg/m^2^)	22.5 ± 1.9	23.6 ± 2.8	26.3 ± 3.0	<0.001
WC (cm)	87.4 ± 7.9	89.6 ± 8.3	95.8 ± 10.2	<0.001
HC (cm)	94.9 ± 5.8	96.6 ± 9.5	100.8 ± 8.1	<0.001
WHR	0.92 ± 0.06	0.93 ± 0.06	0.95 ± 0.06	<0.001
TG (mmol/L)	1.7 ± 1.1	1.6 ± 1.0	2.6 ± 2.1	<0.001
TC (mmol/L)	4.5 ± 1.2	4.5 ± 1.2	4.9 ± 1.8	0.001
HDL-c (mmol/L)	1.13 ± 0.28	1.12 ± 0.27	1.08 ± 0.45	0.013
LDL-c (mmol/L)	2.8 ± 1.0	2.9 ± 1.2	3.0 ± 1.1	0.121
Lipoprotein(a) (mg/dL)	12.7 (7.5–24.9)	13.1 (7.9–25.9)	12.4 (5.9–23.7)	0.068
ALT (IU/L)	26.4 ± 17.1	25.1 ± 17.3	30.8 ± 18.2	<0.001
AST (IU/L)	23.6 ± 10.8	22.5 ± 10.6	25.1 ± 11.9	0.002
GGT (U/L)	22 (16–34)	20 (15–27)	28 (20–43)	<0.001
UA (μmol/L)	281.7 ± 93.8	282.2 ± 94.2	306.7 ± 96.6	0.001
HbA_1c_ (%)	8.5 ± 2.2	8.5 ± 2.2	8.6 ± 1.9	0.201
Smoking history (%)	139 (25.9%)	70 (22.8%)	69 (30.1%)	0.110
Alcohol consumption history (%)	137 (25.6%)	64 (20.8%)	73 (31.9%)	0.009
Hypertension (%)	323 (60.3%)	179 (58.3%)	144 (62.9%)	0.284
Hyperlipidemia (%)	333 (62.1%)	166 (54.1%)	167 (72.9%)	<0.001
Central obesity (%)	391 (72.9%)	197 (64.2%)	194 (84.7%)	<0.001
MetS (%)	423 (78.9%)	222 (72.3%)	201 (87.8%)	<0.001
T2DM duration (years)	10.3 ± 7.5	11.4 ± 7.3	8.1 ± 6.5	<0.001
DR (%)	247 (46.1%)	158 (51.5%)	89 (38.9%)	<0.001
DPN (%)	300 (56.0%)	190 (61.9%)	110 (48.0%)	0.002

Data are expressed as mean ± SD (variables with a normal distribution) or median (25–75%) (variables with a non-normal distribution). SBP, Systolic blood pressure; DBP, Diastolic blood pressure; BMI, Body mass index; WC, Waist circumference; HC, hip circumference; WHR, waist-to-hip ratio; TG, Triglycerides; TC, Total cholesterol; HDL, High-density lipoprotein; LDL, Low-density lipoprotein; ALT, Alanine aminotransferase; AST, Aspartate aminotransferase; GGT, γ-glutamyltransferase; UA, uric acid; HbA_1c_, hemoglobin A1c; MetS, metabolic syndrome; T2DM, Type 2 diabetes mellitus; DR, diabetic retinopathy; DPN, diabetic peripheral neuropathy.

Obesity is strongly associated with NAFLD, which can also be observed in non-obese individuals. Although literature data indicate that NAFLD patients with a normal BMI have their own metabolic characteristics such as more subcutaneous fat, higher levels of triglycerides, lower fasting glucose, less advanced necro-inflammatory activity, and fibrosis compared with obese NAFLD subjects (17), data on long-term survival and mortality are insufficient and controversial. We explored the difference in clinical characteristics between obese MAFLD (BMI ≥ 25 kg/m^2^) and non-obese MAFLD (BMI < 25 kg/m^2^) in T2DM patients (20). The prevalence of MAFLD was 63.0%, in obese subjects and 25.5% in lean subjects. As shown in Table 2, obese individuals with MAFLD had higher systolic blood pressure, BMI, waist circumference, waist-to-hip ratio, triglyceride, total cholesterol, ALT, AST, and GGT levels, a younger age, higher prevalence of hyperlipidemia, a shorter duration of T2DM, and a lower incidence of diabetic retinopathy than those without MAFLD (*p* < 0.05). Non-obese individuals with MAFLD had higher BMI, waist circumference, triglyceride, ALT, AST, GGT, uric acid levels, lower systolic blood pressure and high-density lipoprotein cholesterol concentration, younger age, higher prevalence of hyperlipidemia, central obesity, MetS, shorter duration of T2DM, and lower incidence of diabetic retinopathy and diabetic peripheral neuropathy, compared with non-obese participants without MAFLD (*p* < 0.05), and no statistical difference was observed in hip circumference, waist-to-hip ratio, or total cholesterol between the two groups. It is worth noting that compared with the obese MAFLD group, non-obese MAFLD patients had lower systolic blood pressure, low-density lipoprotein cholesterol levels, and shorter duration of T2DM, not to mention lower BMI, waist circumference, hip circumference, and a lower incidence of central obesity (*p* < 0.05).

**Table 2:** Comparison of obese MAFLD and non-obese MAFLD.

Characteristics	Obese			Non-obese			*P* (O vs. N)
	Without MAFLD	With MAFLD (O)	*P*	Without MAFLD	With MAFLD (N)	*P*	
N	91	155		216	74		
Sex (female subjects/male subjects)	21/70	46/109	0.262	83/133	31/43	0.598	0.067
Age (years)	58.4 ± 11.8	55.1 ± 12.1	0.040	61.1 ± 12.0	55.9 ± 10.3	0.001	0.609
SBP (mmHg)	131.8 ± 17.9	137.2 ± 17.2	0.024	135.8 ± 28.3	128.1 ± 19.7	0.035	0.001
DBP (mmHg)	79.2 ± 18.0	80.3 ± 10.2	0.615	77.4 ± 13.2	79.7 ± 15.1	0.281	0.724
BMI (Kg/m^2^)	26.9 ± 1.6	27.7 ± 2.4	0.003	22.1 ± 1.9	23.3 ± 1.3	<0.001	<0.001
WC (cm)	95.7 ± 7.1	99.0 ± 10.2	0.012	86.8 ± 8.4	89.0 ± 6.0	0.020	0.035
HC (cm)	102.3 ± 12.4	103.1 ± 7.6	0.578	94.3 ± 6.9	96.0 ± 6.8	0.092	<0.001
WHR	0.94 ± 0.08	1.03 ± 0.60	0.009	0.92 ± 0.06	0.93 ± 0.05	0.257	0.163
TG (mmol/L)	1.7 ± 0.9	2.7 ± 2.2	<0.001	1.6 ± 1.0	2.4 ± 1.9	0.001	0.292
TC (mmol/L)	4.5 ± 1.3	4.9 ± 1.2	0.013	4.5 ± 1.2	4.6 ± 1.1	0.613	0.726
HDL-c (mmol/L)	1.1 ± 0.3	1.1 ± 0.2	0.316	1.15 ± 0.28	1.07 ± 0.26	0.044	0.144
LDL-c (mmol/L)	3.0 ± 1.2	3.1 ± 1.2	0.334	2.8 ± 1.0	2.8 ± 1.0	0.879	0.046
Lipoprotein(a) (mg/dL)	14.6 (8.8–23.5)	11.6 (5.9–21.8)	0.943	12.5 (7.9–28.1)	12.4 (6.3–23.9)	0.096	0.397
ALT (IU/L)	25.5 ± 18.9	30.8 ± 18.4	0.030	24.9 ± 16.7	30.6 ± 17.9	0.014	0.928
AST (IU/L)	21.8 ± 11.2	24.7 ± 11.9	0.049	22.8 ± 10.4	25.8 ± 11.9	0.040	0.512
GGT (U/L)	21.0 (15.0–28.5)	30.0 (21.0–50.0)	0.003	18.0 (14.0–26.0)	25.0 (19.0–40.5)	0.007	0.083
UA (μmol/L)	311.4 ± 91.1	301.7 ± 100.6	0.485	370.7 ± 93.2	317.1 ± 87.4	0.001	0.298
HbA_1c_ (%)	9.4 ± 7.8	8.7 ± 1.8	0.387	8.9 ± 5.8	8.6 ± 2.1	0.886	0.763
Smoking history	33 (36.2%)	49 (31.6%)	0.455	37 (17.1%)	20 (27.0%)	0.064	0.479
Alcohol consumption history (%)	22 (24.2%)	54 (34.8%)	0.081	42 (19.4%)	19 (25.7%)	0.256	0.875
Hypertension	51 (56.0%)	103 (66.5%)	0.103	128 (59.3%)	41 (55.4%)	0.562	0.106
Hyperlipidemia	49 (53.8%)	111 (71.6%)	<0.001	117 (54.2%)	56 (75.7%)	0.001	0.518
Central obesity	81 (89.0%)	143 (92.3%)	0.389	116 (53.7%)	51 (68.9%)	0.022	0.048
MetS	83 (91.2%)	140 (90.3%)	0.309	139 (64.4%)	61 (82.4%)	0.004	0.080
T2DM duration (years)	11.4 ± 6.4	8.7 ± 6.8	0.005	11.5 ± 7.7	6.7 ± 5.9	<0.001	0.034
DR	52 (57.1%)	66 (42.6%)	0.027	106 (49.1%)	23 (31.1%)	0.007	0.095
DPN	56 (61.5%)	77 (49.7%)	0.072	134 (62.0%)	33 (44.6%)	0.009	0.472

Data are expressed as mean ± SD (variables with a normal distribution) or median (25–75%) (variables with a non-normal distribution). O, obese; N, non-obese; SBP, Systolic blood pressure; DBP, Diastolic blood pressure; BMI, Body mass index; WC, Waist circumference; HC, Hip circumference; WHR, Waist-to-hip ratio; TG, Triglycerides; TC, Total cholesterol; HDL, High-density lipoprotein; LDL, Low-density lipoprotein; ALT, Alanine aminotransferase; AST, Aspartate aminotransferase; GGT, γ-glutamyltransferase; UA, uric acid; HbA_1c_, hemoglobin A1c; MetS, metabolic syndrome; T2DM, Type 2 diabetes mellitus; DR, diabetic retinopathy; DPN, diabetic peripheral neuropathy.

**Figure 1:**
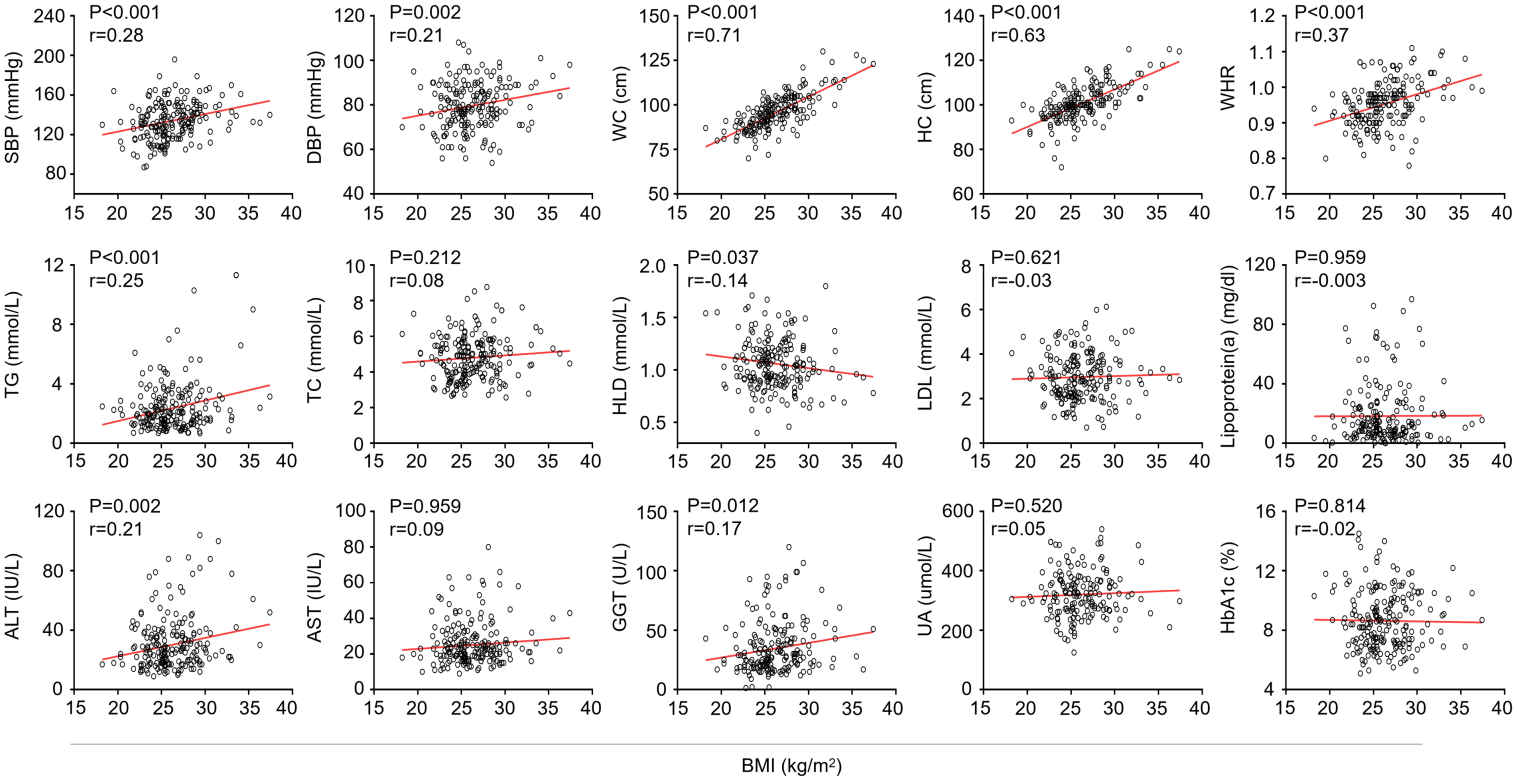
The relationship between BMI and variables. The association of BMI with variables in T2DM patients with MAFLD was assessed by linear regression analysis. SBP, Systolic blood pressure; DBP, Diastolic blood pressure; WC, Waist circumference; HC, Hip circumference; WHR, Waist-to-hip ratio; TG, Triglyceride; TC, Total cholesterol; HDL, High-density lipoprotein; LDL, Low-density lipoprotein; ALT, Alanine aminotransferase; AST, Aspartate aminotransferase; GGT, γ-glutamyltransferase; UA, Uric acid; HbA_1c_, Hemoglobin A1c.

To gain a deeper understanding of the relationship between BMI and variables in T2DM patients with MAFLD, linear regression analysis was applied. The results showed that BMI was significantly and positively associated with systolic blood pressure, diastolic blood pressure, waist circumference, hip circumference, waist-to-hip ratio, triglycerides, ALT, and GGT, and negatively associated with high-density lipoprotein cholesterol (Figure 1).

### Correlation between risk factors and MAFLD in T2DM patients

Univariate logistic regression models were performed to test the potential relationship between variables and the ORs of MAFLD. In the total participants, BMI, waist circumference, hip circumference, waist-to-hip ratio, triglycerides, total cholesterol, ALT, AST, GGT, uric acid, proportion of alcohol consumption, hyperlipidemia, central obesity, and MetS were found to be positively associated with the ORs of MALFD, and there was a negative association of age, high-density lipoprotein cholesterol, and duration of T2DM with the ORs of MALFD (Table 3). Meanwhile, BMI, waist circumference, triglyceride, ALT, AST, GGT, and the incidence of hyperlipidemia were positively correlated with the ORs of MALFD; age and duration of T2DM were negatively correlated with the ORs of MALFD in both obese and non-obese subjects (Table 3). In obese participants, waist-to-hip ratio and total cholesterol were also found to be positively associated with the ORs of MALFD. In non-obese subjects, uric acid and the incidence of central obesity and MetS were also found to be positively associated with the ORs of MALFD; high-density lipoprotein cholesterol was also found to be positively associated with the ORs of MALFD. It is worth noting that higher systolic blood pressure was a risk factor for MAFLD in obese subjects but was negatively associated with the ORs of MALFD in non-obese subjects.

**Table 3:** Univariate logistic regression examined the risk factors for MAFLD.

Characteristics	Total		Obese		Non-obese	
	OR (95% CI)	*P*	OR (95% CI)	*P*	OR (95% CI)	*P*
Sex (female subjects/male subjects)	0.99 (0.69–1.42)	0.951	1.41 (0.77–2.56)	0.263	1.16 (0.68–1.98)	0.598
Age (years)	0.61 (0.44–0.87)	0.006	0.98 (0.96–0.99)	0.042	0.54 (0.31–0.92)	0.022
SBP (mmHg)	1.25 (0.88–1.78)	0.205	2.17 (1.26–3.71)	0.005	0.48 (0.27–0.84)	0.010
DBP (mmHg)	1.31 (0.92–1.86)	0.130	1.12 (0.66–1.91)	0.664	1.22 (0.71–2.08)	0.469
BMI (Kg/m^2^)	4.99 (3.44–7.25)	<0.001	2.39 (1.29–4.46)	0.006	3.65 (1.95–6.83)	<0.001
WC (cm)	2.78 (1.92–4.03)	<0.001	1.95 (1.11–3.43)	0.012	1.89 (1.08–3.30)	0.027
HC (cm)	3.19 (2.21–4.61)	<0.001	1.41 (0.82–2.42)	0.215	1.65 (0.94–2.91)	0.083
WHR	1.95 (1.36–2.80)	<0.001	2.52 (1.43–4.46)	0.001	0.93 (0.54–1.61)	0.789
TG (mmol/L)	2.78 (1.94–3.94)	<0.001	3.86 (2.04–7.31)	<0.001	3.65 (2.09–6.37)	<0.001
TC (mmol/L)	1.45 (1.03–2.05)	0.035	1.29 (1.03–1.62)	0.026	1.29 (0.76–2.19)	0.342
HDL-c (mmol/L)	0.62 (0.44–0.88)	0.007	0.98 (0.59–1.66)	0.951	0.35 (0.13–1.98)	0.046
LDL-c (mmol/L)	1.06 (0.75–1.49)	0.755	1.20 (0.71–2.03)	0.490	0.80 (0.47–1.37)	0.419
Lipoprotein(a) (mg/dL)	0.86 (0.60–1.22)	0.394	1.13 (0.63–2.02)	0.687	0.86 (0.50–1.50)	0.597
ALT (IU/L)	2.31 (1.62–3.29)	<0.001	1.90 (1.06–3.40)	0.030	1.79 (1.05–3.06)	0.032
AST (IU/L)	1.61 (1.14–2.28)	0.007	2.09 (1.15–3.80)	0.015	2.00 (1.17–3.41)	0.011
GGT (U/L)	3.38 (2.34–4.90)	<0.001	3.46 (1.97–6.08)	<0.001	2.31 (1.31–4.01)	0.004
UA (μmol/L)	1.67 (1.16–2.42)	0.006	0.93 (0.53–1.62)	0.791	2.27 (1.26–4.09)	0.006
HbA_1c_ (%)	1.41 (0.99–1.98)	0.053	1.55 (0.91–2.66)	0.108	0.98 (0.58–1.67)	0.943
Smoking history (%)	1.09 (0.89–1.34)	0.418	0.79 (0.59–1.06)	0.114	1.18 (0.84–1.64)	0.338
Alcohol consumption history (%)	1.72 (1.14–2.58)	0.009	1.54 (0.85–2.81)	0.155	1.40 (0.73–2.69)	0.308
Hypertension (%)	1.20 (0.85–1.71)	0.303	1.24 (0.69–2.25)	0.474	0.85 (0.50–1.46)	0.562
Hyperlipidemia (%)	2.30 (1.58–3.33)	<0.001	2.19 (1.28–3.77)	0.005	2.56 (1.40–4.67)	0.002
Central obesity (%)	3.23 (2.06–5.05)	<0.001	1.53 (0.61–3.87)	0.365	1.97 (1.10–3.52)	0.023
MetS (%)	2.72 (1.70–4.35)	<0.001	0.90 (0.37–2.23)	0.826	2.59 (1.34–5.02)	0.005
T2DM duration (years)	0.41 (0.29–0.59)	<0.001	0.38 (0.22–0.66)	0.001	0.26 (0.13–0.50)	<0.001

Data are presented as odds ratios (OR) with 95% confidence intervals (95% CI). BMI, Body mass index; WC, Waist circumference; HC, Hip circumference; WHR, Waist-to-hip ratio; TG, Triglycerides; TC, Total cholesterol; HDL, High-density lipoprotein; ALT, Alanine aminotransferase; AST, Aspartate aminotransferase; GGT, γ-glutamyltransferase; UA, uric acid.

Multivariable logistic regression analyses showed that BMI, hip circumference, triglycerides, ALT, AST, GGT, uric acid, HbA1c, and the incidence of alcohol consumption, hyperlipidemia, central obesity, and MetS were found to be positively associated with the ORs of MALFD, and there was a negative association of the duration of T2DM with the ORs of MALFD in the total participants (Table 4). In obese participants, systolic blood pressure, BMI, waist-to-hip ratio, ALT, AST, triglyceride, and GGT were found to be positively associated with the ORs of MALFD; however, the incidence of smoking history and duration of T2DM were found to be negatively associated with the ORs of MALFD (Table 4). In non-obese participants, BMI, triglycerides, AST, uric acid, and proportion of hyperlipidemia remained positively associated with the ORs of MALFD; T2DM duration remained negatively associated with the ORs of MALFD, which was analyzed by multivariable logistic regression (Table 4).

**Table 4:** Multivariable logistic regression explored the risk factors of MAFLD.

Characteristics	Total		Obese		Non-obese	
	OR (95% CI)	*P*	OR (95% CI)	*P*	OR (95% CI)	*P*
Sex (female subjects/male subjects)	1.18 (0.70–1.99)	0.537	1.02 (0.46–2.26)	0.971	1.73 (0.76–3.95)	0.190
Age (years)	0.89 (0.57–1.40)	0.622	1.14 (0.58–2.25)	0.706	0.59 (0.30–1.16)	0.127
SBP (mmHg)	1.20 (0.77–1.86)	0.418	1.98 (1.04–3.79)	0.039	0.51 (0.25–1.05)	0.069
DBP (mmHg)	1.21 (0.78–1.87)	0.388	1.16 (0.61–2.19)	0.652	0.76 (0.39–1.47)	0.411
BMI (Kg/m^2^)	5.12 (3.32–7.91)	<0.001	2.18 (1.09–4.37)	0.028	5.40 (2.46–11.9)	<0.001
WC (cm)	1.19 (0.69–2.05)	0.525	1.80 (0.92–3.53)	0.086	1.38 (0.66–2.86)	0.390
HC (cm)	2.28 (1.36–3.81)	0.002	1.17 (0.59–2.31)	0.661	1.40 (0.68–2.86)	0.364
WHR	1.28 (0.81–2.03)	0.298	1.93 (1.01–3.69)	0.048	0.72 (0.36–1.42)	0.341
TG (mmol/L)	2.28 (1.48–3.51)	<0.001	3.21 (1.51–6.81)	0.002	3.88 (1.96–7.70)	<0.001
TC (mmol/L)	1.23 (0.80–1.87)	0.344	1.20 (0.64–2.28)	0.567	1.90 (0.98–3.70)	0.058
HDL-c (mmol/L)	0.71 (0.46–1.09)	0.115	1.05 (0.57–1.95)	0.868	0.75 (0.39–1.46)	0.401
LDL-c (mmol/L)	0.91 (0.59–1.38)	0.650	0.99 (0.53–1.84)	0.972	0.94 (0.49–1.81)	0.845
Lipoprotein(a) (mg/dL)	0.80 (0.52–1.24)	0.326	0.77 (0.39–1.53)	0.460	1.04 (0.54–2.02)	0.904
ALT (IU/L)	2.86 (1.84–4.44)	<0.001	2.35 (1.15–4.81)	0.019	1.92 (1.00–3.72)	0.051
AST (IU/L)	2.20 (1.42–3.41)	<0.001	2.83 (1.41–5.68)	0.003	2.43 (1.26–4.70)	0.008
GGT (U/L)	3.04 (1.92–4.80)	<0.001	3.90 (1.98–7.67)	<0.001	1.68 (0.82–3.42)	0.156
UA (μmol/L)	1.70 (1.07–2.71)	0.025	0.78 (0.40–1.53)	0.470	2.93 (1.42–6.06)	0.004
HbA_1c_ (%)	1.53 (1.00–2.34)	0.049	1.30 (0.69–2.44)	0.421	1.50 (0.76–4.91)	0.242
Current smoker (%)	0.77 (0.56–1.06)	0.110	0.64 (0.41–0.99)	0.044	1.06 (0.65–1.75)	0.812
Alcohol consumption (%) history	1.97 (1.10–3.54)	0.023	2.03 (0.90–4.54)	0.087	1.94 (0.76–4.91)	0.164
Hypertension (%)	1.32 (0.85–2.05)	0.218	1.74 (0.91–3.32)	0.094	0.79 (0.40–1.55)	0.486
Hyperlipidemia (%)	2.09 (1.33–3.27)	0.001	1.46 (0.78–2.75)	0.241	2.81 (1.33–5.96)	0.007
Central obesity (%)	2.37 (1.28–4.39)	0.006	1.50 (0.44–5.13)	0.519	1.95 (0.86–4.41)	0.108
MetS (%)	1.97 (1.09–3.54)	0.025	0.66 (0.22–2.00)	0.459	2.07 (0.90–4.73)	0.087
T2DM duration (years)	0.33 (0.21–0.52)	<0.001	0.36 (0.18–0.69)	0.002	0.24 (0.11–0.50)	<0.001

Data are presented as odds ratio (OR) with a 95% confidence interval (95% CI). Adjusted for age, sex, BMI, HbA1c, history of smoking, history of alcohol consumption, and T2DM duration. BMI, Body mass index; WC, Waist circumference; HC, Hip circumference; WHR, Waist-to-hip ratio; TG, Triglycerides; TC, Total cholesterol; HDL, High-density lipoprotein; ALT, Alanine aminotransferase; AST, Aspartate aminotransferase; GGT, γ-glutamyltransferase; UA, uric acid.

### The prevalence of diabetic peripheral neuropathy and diabetic retinopathy was negatively correlated with MAFLD

As mentioned above, the incidence of diabetic retinopathy and diabetic peripheral neuropathy was lower in participants with MAFLD compared to those without MAFLD (Tables 1, 2). To test the possible association between diabetic retinopathy, diabetic peripheral neuropathy, and the ORs of MAFLD, univariate logistic regression models were performed (Model 1). The results showed that diabetic retinopathy and diabetic peripheral neuropathy were negatively associated with the ORs of MALFD in all participants (Table 5). After adjustment for model 2 that included age, sex, HbA1c, smoking history, alcohol consumption history, T2DM duration, and BMI, the ORs of MALFD remained significantly associated with diabetic retinopathy (OR 0.58, 95% CI 0.36–0.93, *p* = 0.023) and diabetic peripheral neuropathy (OR 0.57, 95% CI 0.37–0.89, *p* = 0.013), in all participants (Table 5). However, after adjustment for Model 3, which further included systolic blood pressure, diastolic blood pressure, triglycerides, total cholesterol, high-density lipoprotein cholesterol, low-density lipoprotein cholesterol, ALT, AST, GGT, and uric acid, the ORs of NAFLD were not significantly associated with diabetic retinopathy (OR 0.60, 95% CI 0.34–1.06, *p* = 0.080) and diabetic peripheral neuropathy (OR 0.60, 95% CI 0.35–1.05, *p* = 0.071) (Table 5).

**Table 5:** Odds ratios of MALFD in T2DM patients—univariate and multivariate associations with DR and DPN.

Characteristics	Total		Obese		Non-obese	
	OR (95% CI)	*P*	OR (95% CI)	*P*	OR (95% CI)	*P*
DR						
Model 1	0.60 (0.41–0.86)	0.006*	0.54 (0.31–0.94)	0.028*	0.47 (0.26–0.86)	0.014*
Model 2	0.58 (0.36–0.93)	0.023*	0.77 (0.39–1.49)	0.433	0.43 (0.20–0.93)	0.032*
Model 3	0.60 (0.34–1.06)	0.080	0.84 (0.37–1.88)	0.663	0.28 (0.09–0.85)	0.025*
DPN						
Model 1	0.56 (0.39–0.81)	0.002*	0.56 (0.34–0.90)	0.067	0.50 (0.29–0.86)	0.012*
Model 2	0.57 (0.37–0.89)	0.013*	0.57 (0.30–1.09)	0.087	0.65 (0.34–1.27)	0.209
Model 3	0.60 (0.35–1.05)	0.071	0.58 (0.26–1.30)	0.184	0.64 (0.26–1.57)	0.329

Data are expressed as odds ratio (OR) with a 95% confidence interval (95% CI). DR, diabetic retinopathy; DPN, diabetic peripheral neuropathy. Model 1: not adjusted for any factor. Model 2: adjusted for age, sex, BMI, HbA1c, history of smoking, history of alcohol consumption, and T2DM duration. Model 3: adjusted for model 2 plus SBP, DBP, TG, TC, HDL-C, LDL-C, ALT, AST, GGT, and UA. *Statistically significant *p*-values.

It is worth noting that, in non-obese participants, after adjustment for confounding factors such as age, sex, HbA1c, smoking history, T2DM duration, BMI, systolic blood pressure, diastolic blood pressure, triglycerides, total cholesterol, high-density lipoprotein cholesterol, low-density lipoprotein cholesterol, ALT, AST, GGT, and uric acid, the ORs of MALFD remained negatively associated with diabetic retinopathy but not with diabetic peripheral neuropathy (Table 5). However, in obese participants, after adjustment for all these confounding factors, the ORs of NAFLD were not significantly associated with diabetic retinopathy or diabetic peripheral neuropathy (Table 5). This suggested that there were clinicopathological differences between obese and non-obese MAFLD in T2DM patients.

## Discussion

MAFLD represents an important burden of disease for patients with T2DM; however, the magnitude of the problem is currently unknown. In this study, the prevalence of MAFLD in hospitalized Chinese T2DM patients was calculated to be 42.7%, which is slightly higher than the 42.1% prevalence of NAFLD in Chinese T2DM patients in a previous report (21), but lower than the prevalence (55.5%) reported in a systematic review and meta-analysis of the global epidemiology of NAFLD in T2DM (4). Obesity is strongly associated with NAFLD. However, it can also be observed in non-obese individuals and has its own metabolic characteristics. In the current study, we classified the T2DM patients with MAFLD into non-obese and obese MAFLD (BMI < 25 kg/m^2^, BMI ≥ 25 kg/m^2^). The prevalence of MAFLD was 63.0% in obese subjects and 25.5% in non-obese subjects. Non-obese NAFLD has been characterized as a unique phenotype with specific genetic associations (22). A summary of studies on NAFLD stated that the features of lean NAFLD are different from country to country (23). Thus, it is difficult to detect and treat in its early stages. It has been proven to be an unrecognized clinicopathologic entity and a frequent cause of cryptogenic liver disease (17); therefore, more efforts should be made to halt or reverse the progression of NAFLD in non-obese individuals (18).

In our study, MAFLD was characterized by the presence of significantly higher BMI and triglycerides than non-MAFLD controls in both non-obese and obese participants with T2DM. Even in non-obese patients with T2DM, high BMI was found to be an independent risk factor for MAFLD. A high-fat diet is closely related to a fatty liver; normal hepatocytes contain about 4–7% of lipids, of which triglycerides account for about 1/2. Fatty liver can be caused when the content of triglycerides in the liver increases. It reminded us that reasonable dietary and physical activity status should also be advocated in non-obese subjects with T2DM, even if their BMI is <25 kg/m^2^, as this is helpful in preventing the development of MAFLD. This is supported by a previous report showing the benefit of weight reduction in lean NAFLD patients (24). Also, attention should be paid to monitoring MAFLD in these lean subjects in favor of early intervention. BMI has been reported to have a good performance in predicting NAFLD in the general population (25). Considering the simplicity and convenience of its measurement and without requiring additional laboratory measurements, BMI should be used as an important marker for predicting MAFLD to screen general T2DM patients, especially those non-obese subjects with T2DM.

Waist circumference is a surrogate for visceral obesity that can be used to measure hepatic lipid content (26). Several other studies reported that, compared with controls in the same weight group, both overweight-obese and lean NAFLD patients had higher levels of waist circumference (27). Our results were consistent with theirs.

ALT, AST, and GGT are liver enzymes that are sensitive to hepatic injury, and large amounts of studies showed that elevated hepatic enzymes are manifestations of NAFLD. In addition, serum ALT and GGT concentrations are correlated with the incidence of NAFLD (28). Our study revealed that ALT, AST, and GGT were independent risk factors for MAFLD in obese patients with T2DM. However, in non-obese participants, it was only for ALT and AST, which supports the unique metabolic characteristics of non-obese MAFLD. Considering that research on non-obese NAFLD in T2DM patients is rare, the mechanisms need to be examined more in future studies.

An increasing body of data suggests a link between uric acid and NAFLD. Many recent observations have been made in order to clarify this association (29–31). It can be said that this connection is strengthened by studies that used xanthine oxidase inhibitors in animals to inhibit uric acid production. These studies concluded that the use of xanthine oxidase inhibitors resulted in reduced progression of NAFLD (32–34). Uric acid was found to be an independent risk factor for NAFLD in lean subjects in several other studies conducted in Iran (35) and China (36, 37). However, the relationship between uric acid and NAFLD in obese individuals still needs to be elucidated. Recently, a study of 1,365 obese Chinese adults demonstrated that uric acid was independently and linearly associated with the risk of NAFLD (38). Interestingly, a Brazilian study reached the opposite conclusion: that high levels of uric acid were not associated with NAFLD in overweight or obese children and adolescents (39). Another four-year retrospective cohort study confirmed this, demonstrating that baseline hyperuricemia was positively and significantly associated with NAFLD risk in initially NAFLD-free subjects (36). This relationship was significantly independent of baseline age, sex, metabolic syndrome components, and other clinical variables, but it was found only in non-obese subjects and not in obese subjects. Our results endorsed these findings by showing that uric acid levels were significantly higher in MAFLD patients than in the control group and were positively correlated with MAFLD risk only in non-obese subjects with T2DM but not in obese subjects with T2DM. These observations may be related to the unique metabolic mechanism in non-obese MAFLD, which should be further explored. Uric acid has been reported to be responsible for lipid metabolism impairment and inflammation (40, 41). Thus, there may be an interaction between high serum uric acid and increased weight in the pathogenesis of MAFLD. Hence, all suspected or diagnosed non-obese MAFLD patients, especially those with T2DM, should undergo uric acid testing and specific management for uric acid. Further studies in non-obese MAFLD patients must include uric acid as part of the laboratory tests.

The association between MAFLD and diabetic complications in T2DM has not been thoroughly investigated. Studies from Italy have shown that NAFLD is independently associated with an increased prevalence of chronic kidney disease and proliferative/laser-treated retinopathy in patients with T2DM (13, 42). Research from Romania showed that NAFLD is positively correlated with microalbuminuria, a marker of early-stage nephropathy, in patients with T2DM (43). In contrast to these previous studies, it was reported that NAFLD was inversely associated with the prevalence of diabetic retinopathy and nephropathy in Korean patients with T2DM and was not associated with diabetic neuropathy (44). Other previous studies have shown negative correlations between the prevalence of NAFLD and the duration of diabetes, diabetic retinopathy, diabetic peripheral neuropathy, and diabetic nephropathy (12, 45). Consistent with them, our study revealed that MAFLD was inversely associated with the prevalence of diabetic retinopathy and diabetic peripheral neuropathy in Chinese patients with T2DM. We also found that patients with MAFLD had a significantly shorter duration of T2DM compared with those without MAFLD. This may be a possible explanation for these observations. In addition, it has been speculated that patients with NAFLD may participate in more regular and intense physical activity than non-NAFLD patients to reduce the occurrence of microvascular complications.

Our results confirm and detail the relationship between MAFLD and some of the diabetic complications. It is worth noting that subgroup analysis suggested a difference between obese and non-obese participants. Our results showed that in non-obese patients with T2DM, MAFLD was inversely and independently associated with the prevalence of DR but not with the prevalence of DPN. This relationship was significantly independent of baseline age, sex, HbA1c, history of smoking, history of alcohol consumption, T2DM duration, BMI, systolic blood pressure, diastolic blood pressure, triglycerides, total cholesterol, high-density lipoprotein cholesterol, low-density lipoprotein cholesterol, ALT, AST, GGT, and uric acid. However, in obese patients with T2DM, there was no independently significant relationship between MAFLD and diabetic retinopathy or diabetic peripheral neuropathy. The possible explanation is the different pathophysiology, metabolite profile, and genetic phenotype between overweight-obese and lean patients with MAFLD in T2DM, which is deserving of further investigation.

Our study has several strengths. First, our results confirmed and detailed the relationship between MAFLD and some diabetic complications. The current study is the first to find that in non-obese participants, ORs of MALFD remained negatively associated with diabetic retinopathy but not with diabetic peripheral neuropathy. This association was not found among obese participants. Second, we found that even in non-obese patients with T2DM, high BMI, and triglycerides were independent risk factors for MAFLD, which reminded us that reasonable dietary and physical activity status should also be advocated in subjects with T2DM even if their BMI < 25 kg/m^2^. Third, we found that uric acid levels were positively correlated with MAFLD risk only in non-obese subjects with T2DM but not in obese subjects with T2DM. These observations suggest a unique metabolic mechanism in non-obese MAFLD that should be further explored.

However, our study also has some limitations. First, due to the cross-sectional nature of the study, we cannot establish a causal relationship between MAFLD and risk factors. Second, our study lacks detailed medication data, which may have a potential influence. Third, microvascular complications typically include retinopathy, nephropathy, and neuropathy. Our data on diabetic nephropathy were not sufficient to perform a relevant analysis. Fourth, there was a recall bias for self-reported type 2 diabetes mellitus and hypertension among patients. Finally, there was a lack of detailed data to analyze risk factors for diabetic complications. In a future study, we will also add these data.

In conclusion, the present study assessed the metabolic characteristics of non-obese MAFLD compared with obese MAFLD and the relationship of MAFLD with some diabetic complications in a cohort of patients with T2DM. Even in obese patients with T2DM, BMI was found to be an independent risk factor for MAFLD. These findings support a more structured, risk factor-based approach to MAFLD management, particularly in patients with T2DM. In addition, uric acid was positively correlated with MAFLD risk only in non-obese subjects with T2DM but not in obese subjects with T2DM. We also found that, in non-obese patients with T2DM, there was a negative correlation between the prevalence of MAFLD and diabetic retinopathy. These observations suggest different mechanisms between obese MAFLD and non-obese MAFLD in T2DM patients. Genetic, microbiological, and other metabolic factors warrant further investigation to identify unique determinants of non-obese MAFLD individuals, especially those with T2DM.

## Data availability statement

The raw data supporting the conclusions of this article will be made available by the authors, without undue reservation.

## Ethics statement

The studies involving humans were approved by the Epidemiology Ethics Committee of the second Affiliated Hospital of Xi’an Jiaotong University. The studies were conducted in accordance with the local legislation and institutional requirements. The participants provided their written informed consent to participate in this study.

## Author contributions

S-WD designed the study, analyzed the data, and drafted the manuscript. LG, Y-JL, and RZ collected, interpreted the data, and contributed to the data analysis. JX conceived and supervised the study, revised the manuscript critically for important intellectual content, and supervised the study. All authors approved the manuscript for submission.

## Funding

This work was supported by the National Science Foundation of China (82002990).

## Conflict of interest

The authors declare that the research was conducted in the absence of any commercial or financial relationships that could be construed as a potential conflict of interest.

## Publisher’s note

All claims expressed in this article are solely those of the authors and do not necessarily represent those of their affiliated organizations, or those of the publisher, the editors and the reviewers. Any product that may be evaluated in this article, or claim that may be made by its manufacturer, is not guaranteed or endorsed by the publisher.
